# Supplementary figures and images for: Microglia sense fungal infections through capsular components from capillary-bound Cryptococcus neoformans via endothelial nucleotide signaling
Source: PLoS Biol. 2026 Feb 6;24(2):e3003642. doi: 10.1371/journal.pbio.3003642 (PMC12904584; doi:10.1371/journal.pbio.3003642)

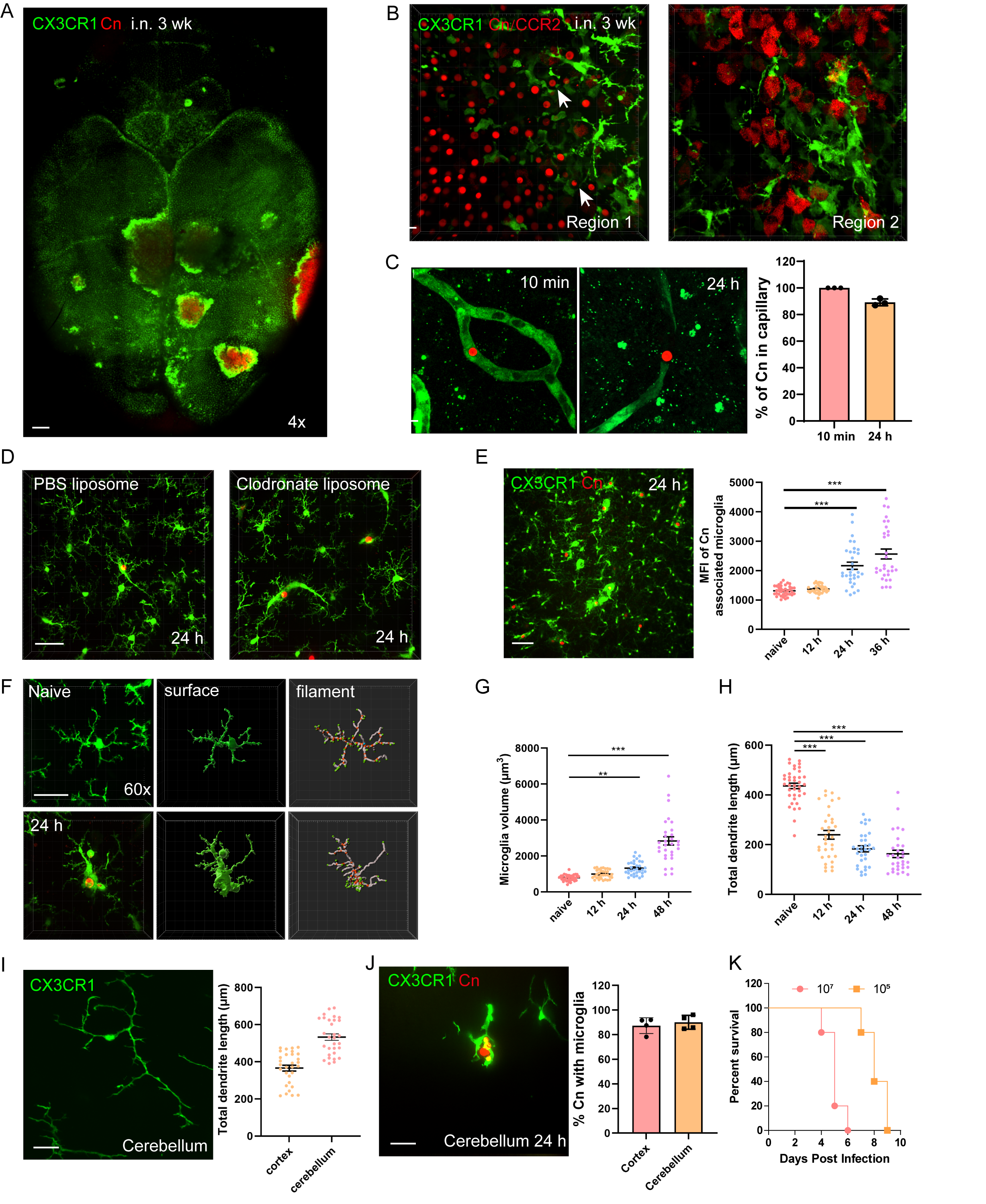

Supplement: S1 Fig — (A) CX3CR1gfp/+ mice were intranasally infected with 1 × 10^4^ tdTomato-labeled C. neoformans H99 strain (H99-tdT) for 3 weeks. The brains were imaged by 4× objective after tissue clearing. (B) CX3CR1gfpCCR2rfp dual reporter mice were i.n. infected with 1 × 10^4^ H99-tdT for 3 weeks. Cleared brains were imaged using a 60× objective. Arrows indicate microglia interaction with fungal cells. (C) Tie2-GFP mice (n = 3 mice/group) were i.v. infected with 1 × 10^7^ H99-tdT for 10 min or 24 h and imaged by whole-mount confocal imaging. Quantification of the percentage of intravascular fungi (right). (D) CX3CR1gfp/+ mice were pretreated with 200 μL PBS liposome or Clodronate liposome for 12 h to deplete monocytes, followed by i.v. infection with 1 × 10^7^ H99-tdT. Microglia recruitment was analyzed 24 h post-infection. (E) CX3CR1gfp/+ mice were i.v. infected with 1 × 10^7^ H99-tdT for indicated time points, the mean fluorescence intensity of microglia was measured for C. neoformans association with microglia (with the exception of naïve). Each dot represents an individual microglial cell pooled from three mice per group. (F) Representative 3D images as well as surface and filament rendering (branching and terminal points are displayed) showing the morphology of microglia (green) with or without association of C. neoformans (red). (G) The volumes of microglia with C. neoformans association at indicated time points. Each dot represents an individual microglia cell pooled from five mice per group. (H) The total dendrite length of microglia with C. neoformans association at indicated time points. Each dot represents an individual microglial cell pooled from five mice per group. (I) Representative image of microglia in the cerebellum (left). Total dendrite length of microglia in the cerebellum compared to those in the cortex (right). (J) Quantification of the percentage of C. neoformans with microglia association in the cerebellum. CX3CR1gfp/+ mice (n = 4 mice/group) were i.v. infected wit [file pbio.3003642.s001.tif]

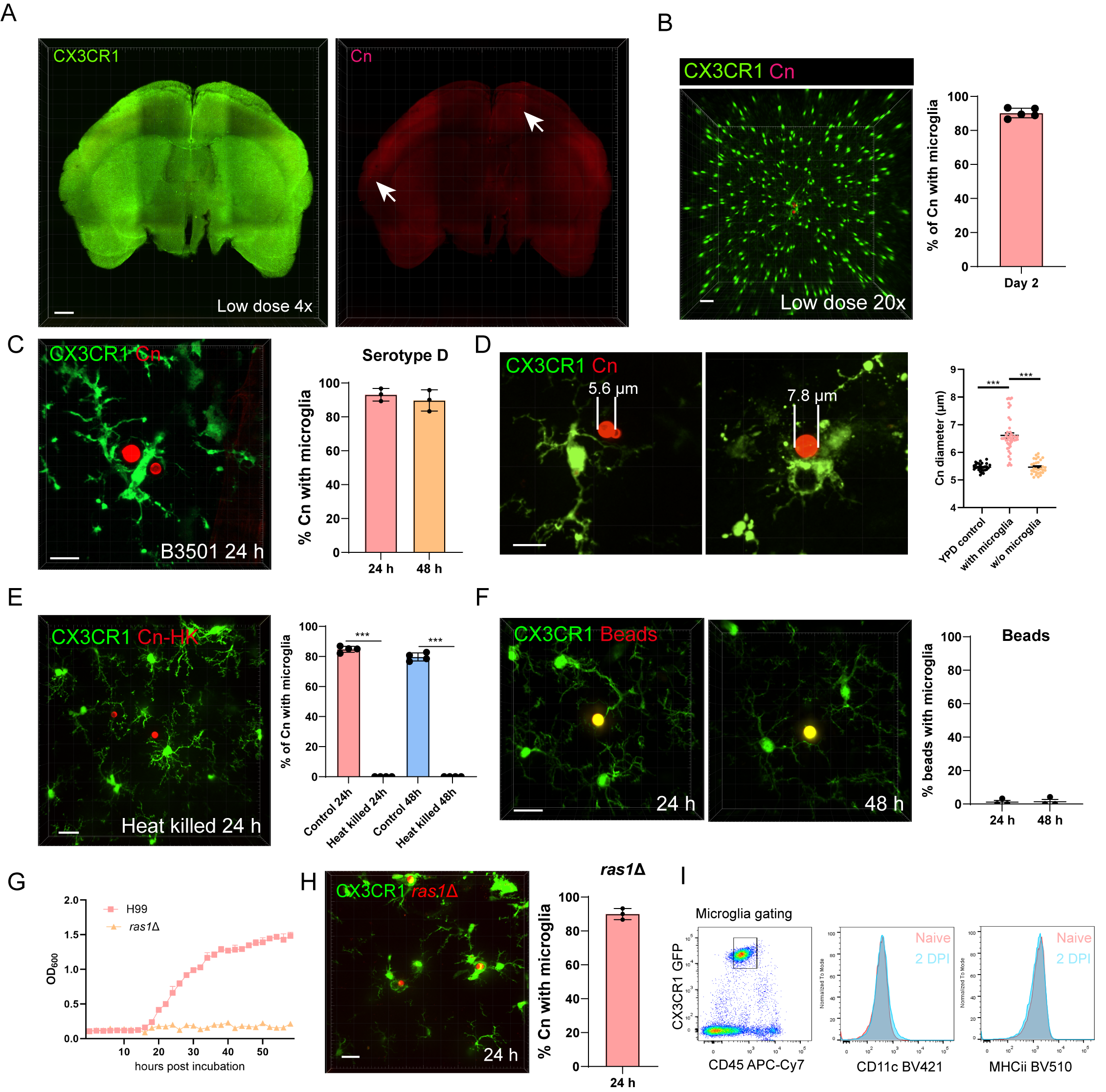

Supplement: S2 Fig — (A) CX3CR1gfp/+ mice were i.v. infected with 1 × 10^5^ tdTomato-labeled Cryptococcus neoformans H99 strain (H99-tdT) for 48 h. The brain slices (1 mm thick) were imaged after tissue clearing, arrows indicate fungal location. (B) Representative image showing the recruitment of microglia to C. neoformans after low dose i.v. infection. Quantification of microglia association with C. neoformans after low dose i.v. infection was shown to the right (n = 5 mice). (C) CX3CR1gfp/+ mice were i.v. infected with 1 × 10^7^ TRITC-labeled B3501 strain for 24 and 48 h. Quantification of microglia association was shown to the right (n = 3 mice/group). (D) CX3CR1gfp/+ mice were i.v. infected with 1 × 10^7^ TRITC-labeled H99 strain for 24 h. The sizes of C. neoformans with or without microglia association were measured (right). Fungi freshly collected from rich medium were included as control. Each dot represents an individual fungus pooled from three mice per group. (E) CX3CR1gfp/+ mice were i.v. infected with 1 × 10^7^ TRITC-labeled heat-killed H99 by 24 and 48 h. Quantification of microglia association was shown to the right (n = 4 mice/group). (F) Representative images showing the relative location of beads with microglia after i.v. infection of 1 × 10^7^ TRITC-labeled polystyrene beads of 8 μm for 24 and 48 h. Quantification of beads association with microglia at 24 and 48 h (right, n = 3 mice/group). (G) The growth of H99 and ras1Δ in YPD medium, confirming the temperature-sensitive property of ras1Δ strain. (H) Representative image showing the association of microglia with ras1Δ mutant strain after i.v. treated with 1 × 10^7^ TRITC-labeled fungi for 24 h. Quantification of microglia association with ras1Δ fungi was shown to the right (n = 3 mice). (I) The analysis of microglial expression of activation makers CD11c and MHCii 2 days after i.v. infection with 1 × 10^7^ H99. Refer to S7C Fig for gating strategy. The data underlying this Figure can be found in S1 Data. Scale bars: 20 μm (A) (B) [file pbio.3003642.s002.tif]

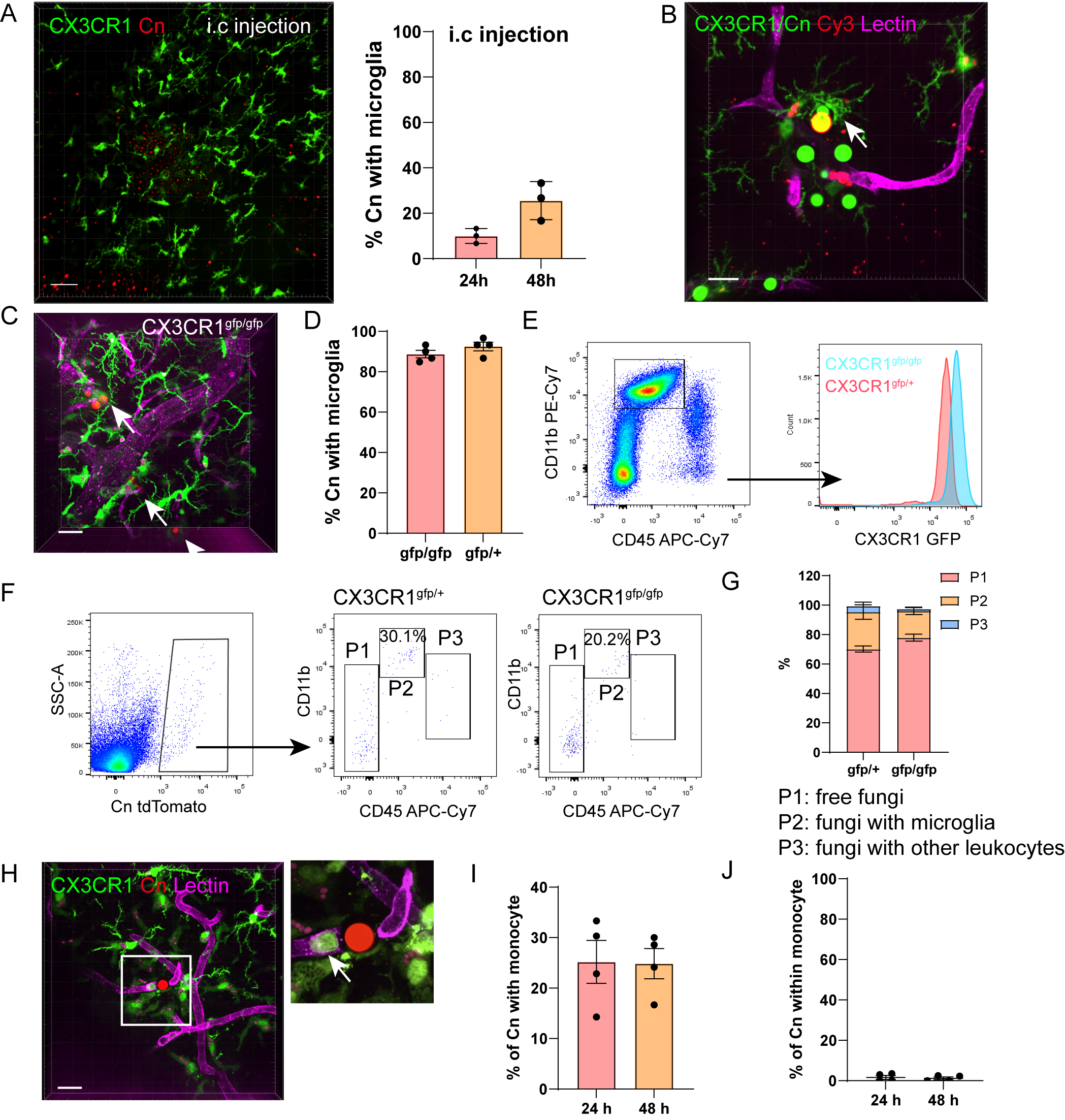

Supplement: S3 Fig — (A) A representative image showing the relative location of microglia after intracerebrally injected with 1 × 10^3^ H99-tdT strain for 48 h (left). Quantification of the percentage of Cryptococcus neoformans with microglia association at indicated time points (right). CX3CR1gfp/+ mice (n = 3 mice/group) were intracerebrally injected with 1 × 10^3^ H99-tdT strain. (B) A representative image showing a microglia cell in association with a daughter cell in a colony with the mother cell already transmigrated the BBB (also see S5 Movie). (C) A representative imaging showing CX3CR1gfp/gfp mice also have microglia association with C. neoformans after i.v. infection with 1 × 10^7^ H99-tdT. (D) Quantification of the percentage of C. neoformans with microglia association in CX3CR1gfp/gfp and CX3CR1gfp/+ mice at indicated time points. Mice (n = 4 mice/group) were i.v. infected with 1 × 10^7^ H99-tdT for 24 and 48 h. The brains were imaged by whole-mount confocal imaging using 60× objective for analysis of microglia association. (E) Flowcytometry identification of microglia in the brain. (F) The association of C. neoformans with microglia and other leukocytes in the brain. CX3CR1gfp/gfp and CX3CR1gfp/+ mice (n = 4 mice/group) were i.v. infected with 1 × 10^7^ H99-tdT for 24 h, the brain leukocytes were isolated and analyzed by flowcytometry. (G) The quantification of C. neoformans in association with microglia and other leukocyte were analyzed by flowcytometry. (H) A representative image showing GFP+ monocyte in close proximity with C. neoformans after i.v. infection with 1 × 10^7^ H99-tdT for 24 h. (I) Quantification of monocyte association with C. neoformans colonies as revealed by in situ imaging. (J) Statistics showing the percentage of C. neoformans being engulfed by monocytes as revealed by in situ imaging. CX3CR1gfp/+ mice (n = 4 mice/group) were i.v. infected with 1 × 10^7^ H99-tdT for 24 and 48 h. The data underlying this Figure can be found in S1 Data. Scale bars: 50 μm (A), 20 μm ( [file pbio.3003642.s003.tif]

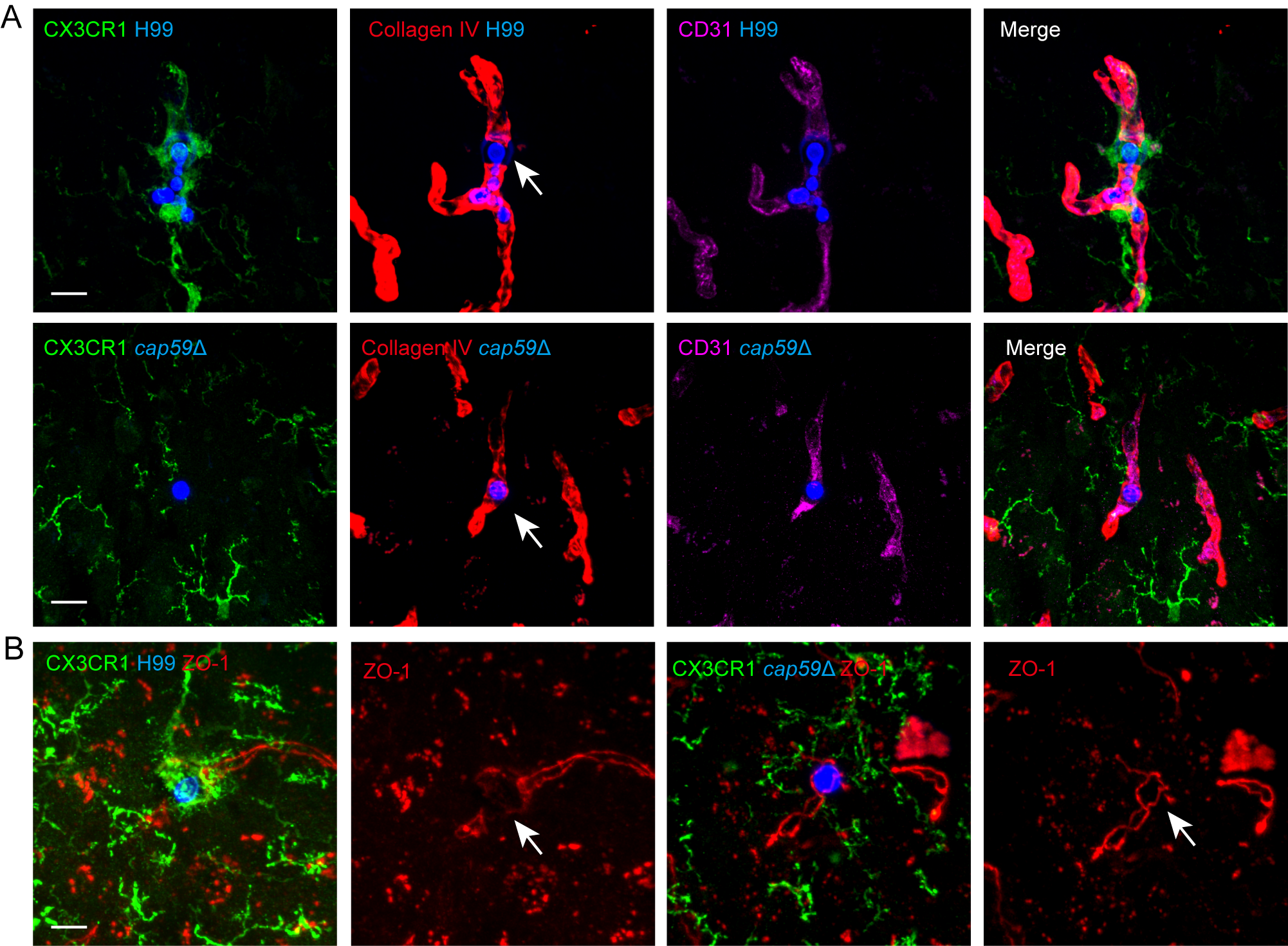

Supplement: S4 Fig — (A) Immunofluorescence images showing the disruption of BBB by wild-type H99 strain as indicated by discontinuous collagen IV and CD31 staining (upper). In contrast, cap59Δ strain showed continuous collagen IV staining (lower). CX3CR1gfp/+ mice were i.v. infected with 1 × 10^7^ Uvitex 2B-labeled fungi for 24 h. (B) Immunofluorescence images showing the disruption of tight junction protein ZO-1 by wild-type H99 strain (left) and cap59Δ strain (right). Scale bar: 20 μm. (TIF) [file pbio.3003642.s004.tif]

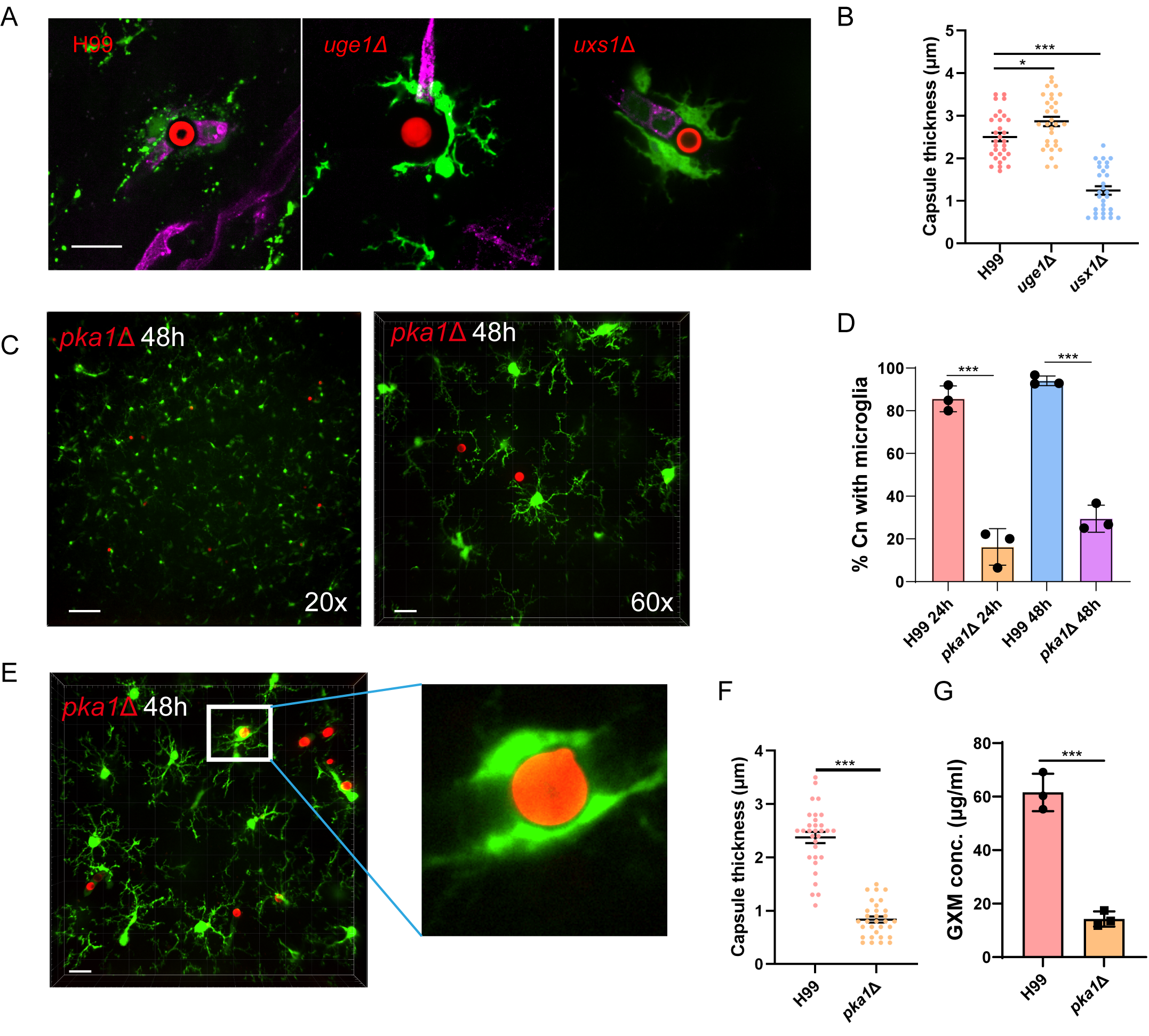

Supplement: S5 Fig — (A) Representative images showing the thickness of fungal strains in the brain. CX3CR1gfp/+ mice were i.v. infected with 1 × 10^7^ TRITC-labeled fungi for 24 h (left). Blood vessels were label by Alexa Fluor 649-conjugated Tomato Lectin (magenta). (B) Quantification of capsule thickness 24 h after i.v. infection. Each dot represents an individual fungal cell pooled from three mice per group. (C) Representative images showing the lack of microglia recruitment for pka1Δ strain, 20× (left), 60× (right). CX3CR1gfp/+ mice were i.v. infected with 1 × 10^7^ TRITC-labeled pka1Δ fungi for 48 h. (D) Quantification of microglia association with pka1Δ strain compared to wild-type H99 strain at indicated time points. (E) Representative images showing the lack of microglia recruitment and reduced capsule size of pka1Δ strain. (F) Quantification of capsule thickness of pka1Δ strain compared to H99. Each dot represents an individual fungal cell pooled from 3 mice per group. (G) Quantification of GXM release in culture medium of H99 and pka1Δ strain. The data underlying this Figure can be found in S1 Data. Scale bar: 10 μm (A), 20 μm (C right) (E), 50 μm (C left), * p < 0.05, *** p < 0.001 by one-way ANOVA (B)(D) or unpaired student t test (F) (G). (TIF) [file pbio.3003642.s005.tif]

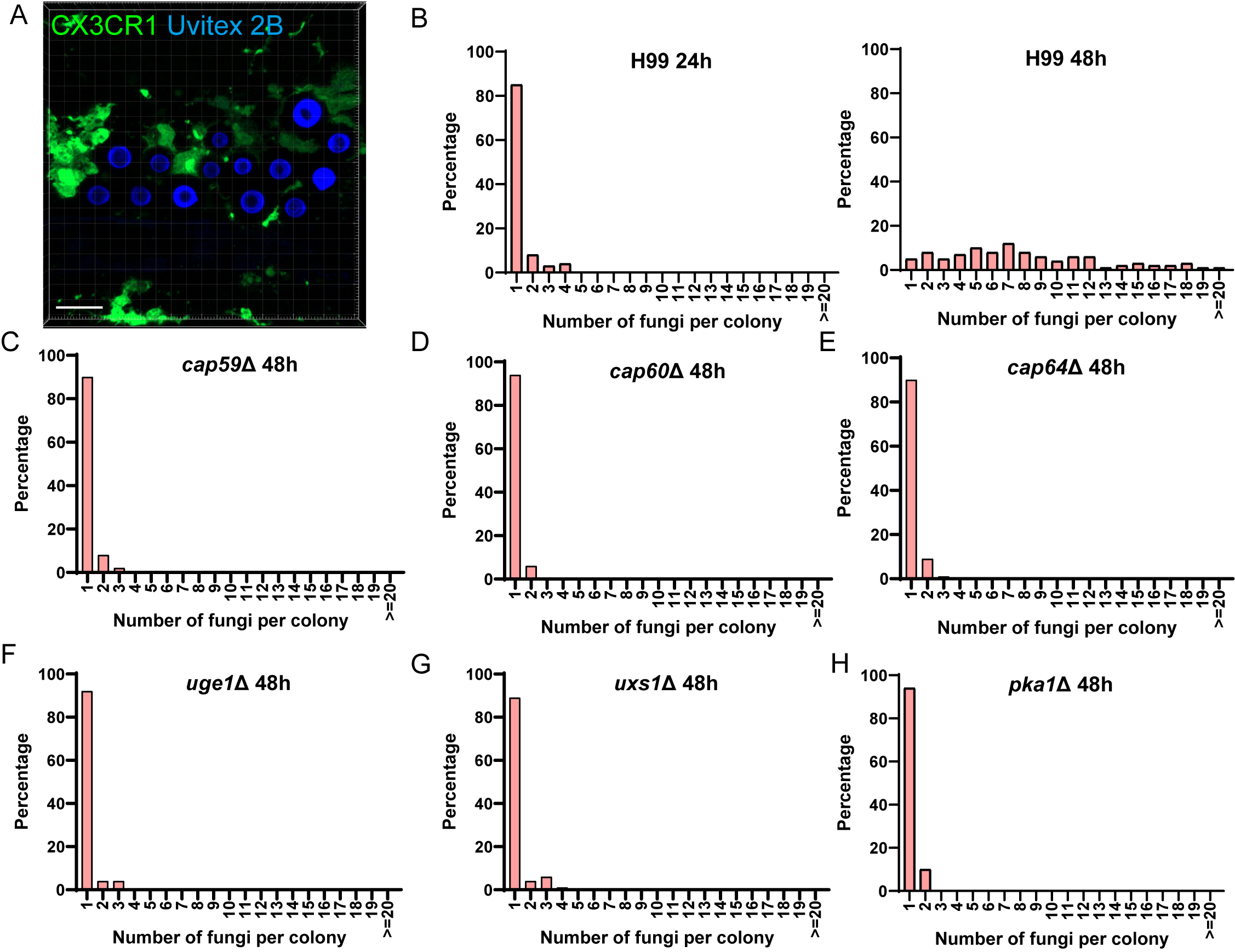

Supplement: S6 Fig — (A) Representative image showing the successful labeling of C. neoformans in the brain by Uvitex 2B injection. Mice were i.v. injected with 100 μL 1% Uvitex 2B was injected through the tail vein 30 min before euthanasia to label C. neoformans in the brain. (B) The number of wild-type H99 cells per colony 24 h (left) or 48 h (right) post infection was enumerated. Mice were i.v. infected with 1 × 10^7^ Uvitex labeled H99 and in vivo labeled by Uvitex 2B. (C–H) The number of fungal cells per colony for indicated strains. Mice were i.v. infected with 1 × 10^7^ Uvitex labeled fungi and analyzed 48 h post-infection. The data underlying this Figure can be found in S1 Data. (TIF) [file pbio.3003642.s006.tif]

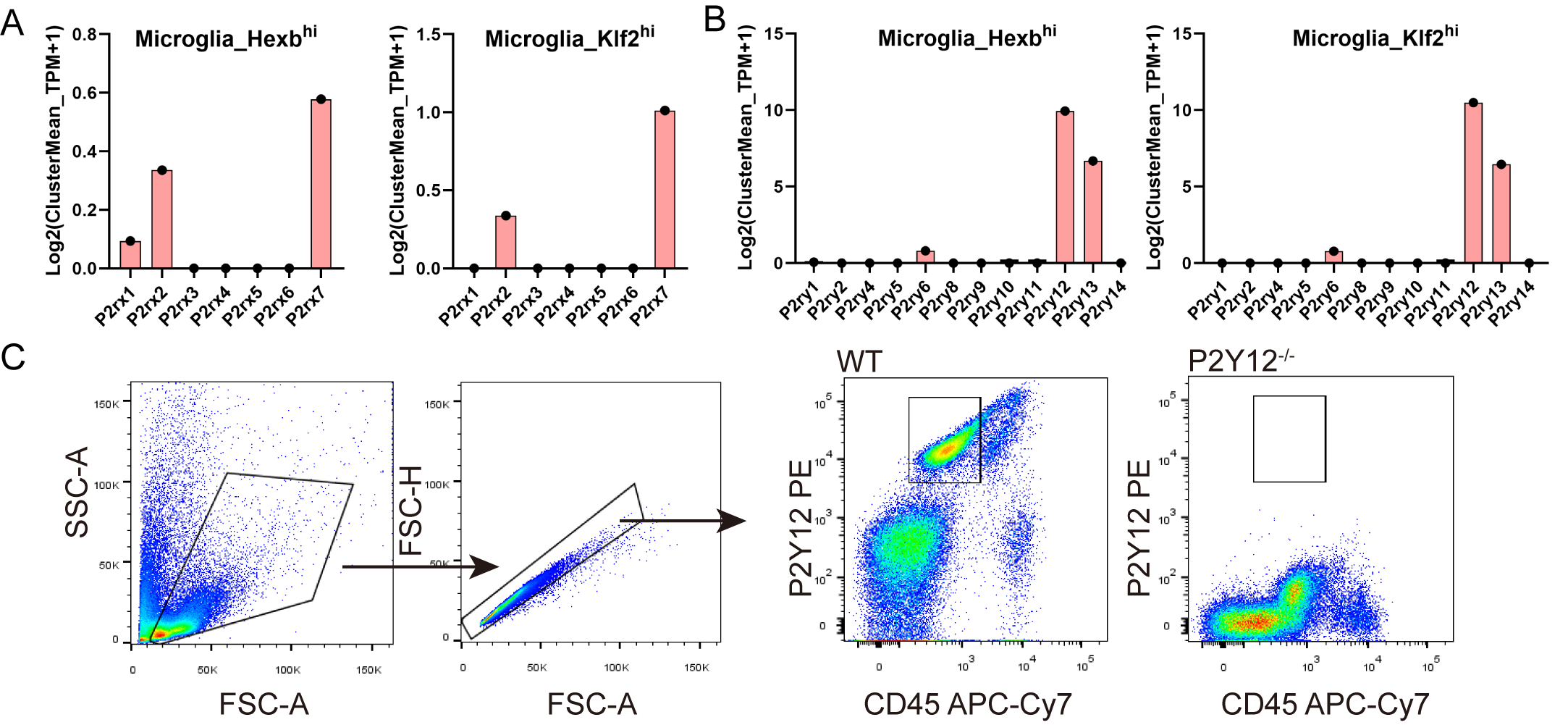

Supplement: S7 Fig — (A) The mRNA of P2X nucleotide receptors detected in microglia (classified as Hexbhi and Klfhi populations). Data obtained from Mouse Cell Atlas (MCA) website at http://bis.zju.edu.cn/MCA/. (B) The mRNA of P2Y nucleotide receptors detected in microglia. (C) Flowcytometry detection of P2Y12 in microglia and the confirmation of successful knockout in P2Y12−/− mice. The data underlying this Figure can be found in S1 Data. (TIF) [file pbio.3003642.s007.tif]

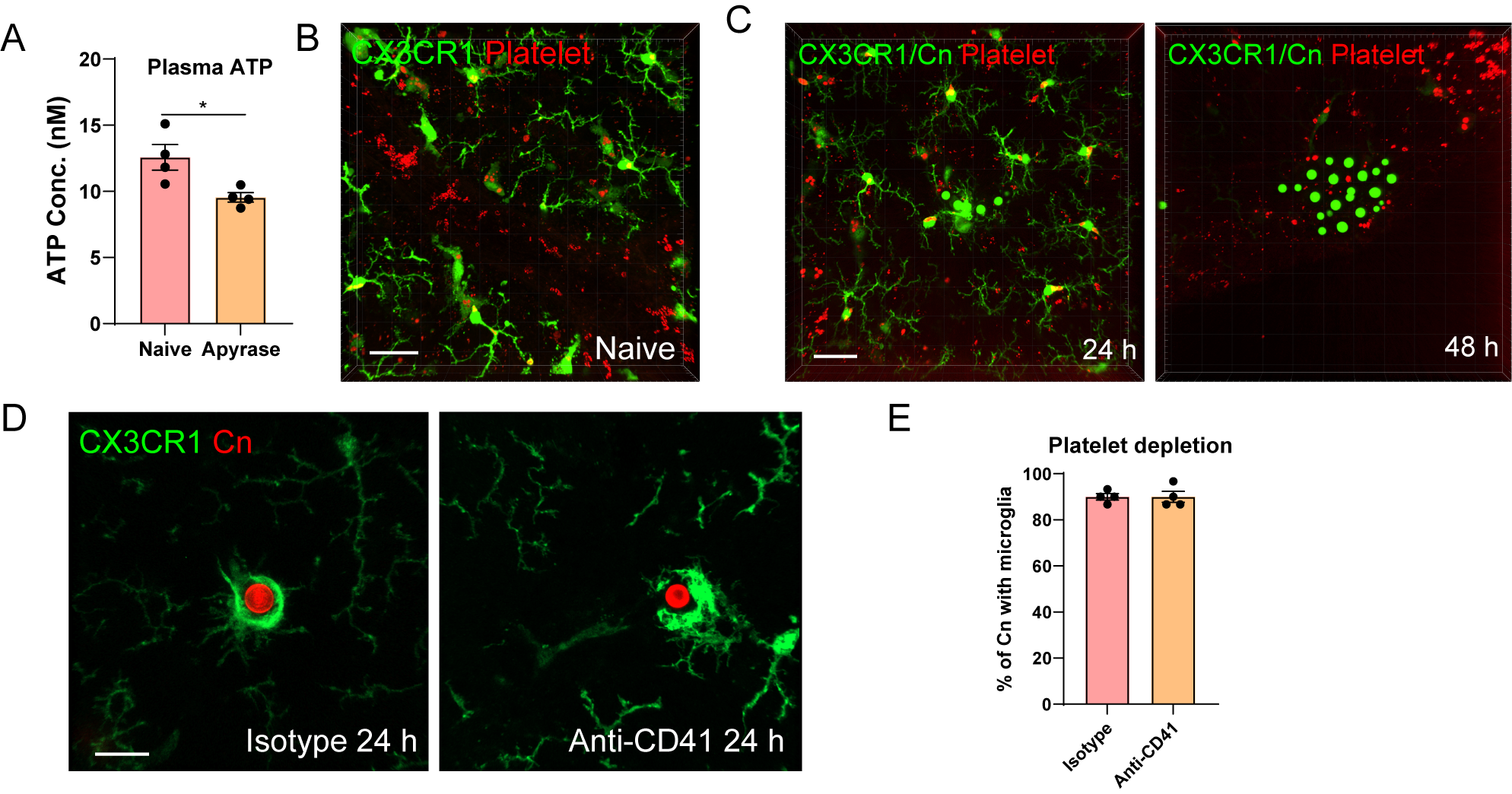

Supplement: S8 Fig — (A) Quantification of ATP levels in the plasma of mice with or without i.v. injection of 25 U/mice apyrase 30 min before transcardial blood collection. (B) Representative images showing the successful labeling of platelet in naïve mice by PE-anti-CD42d antibody. (C) Representative images showing the distribution of platelets in around fungal clusters. CX3CR1gfp/+ mice were i.v. infected with 1 × 10^7^ H99-tdT for 24 h and 48 h, and platelet was labeled by PE-anti-CD42d antibody before euthanizing. (D) Representative images showing the recruitment of microglia to C. neoformans with or without platelet depletion by 200 μg/mice anti-CD41 antibody. CX3CR1gfp/+ mice were i.v. infected with 1 × 10^7^ H99-tdT for 24 h. (E) Quantification of microglia association with C. neoformans with or without platelet depletion 24 h post infection. The data underlying this Figure can be found in S1 Data. Scale bars: 20 μm (B) (C) (D). (TIF) [file pbio.3003642.s008.tif]

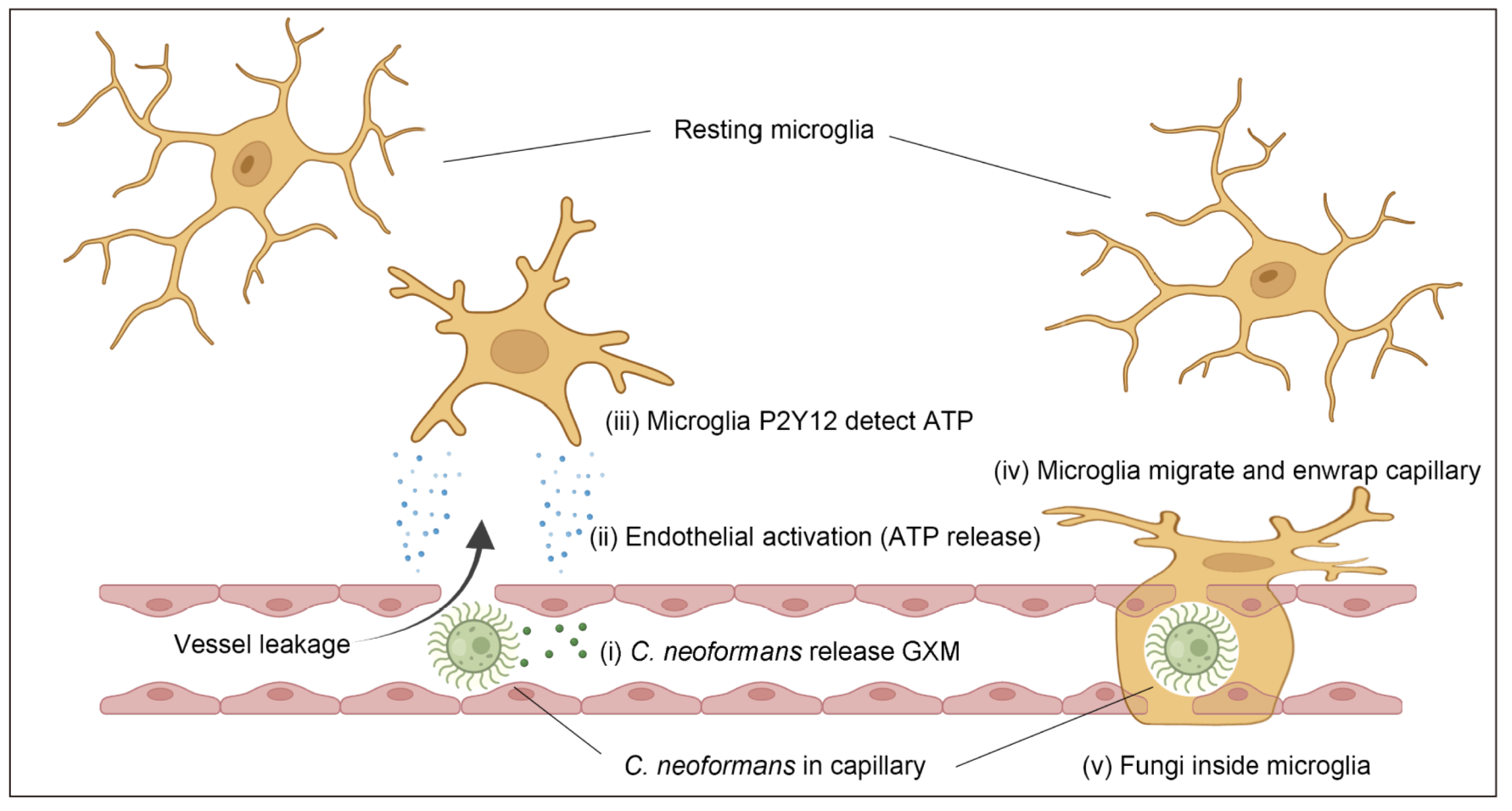

Supplement: S9 Fig — (i) Cryptococcus neoformans trapped in capillaries release GXM. (ii) GXM induces endothelial activation and ATP release. (iii) Microglia P2Y12 detect ATP. (iv) Microglia migrate and enwrap capillary. (v) Microglia engulf C. neoformans and promote its growth. (TIF) [file pbio.3003642.s009.tif]
