# Supplementary material for: Microglia sense fungal infections through capsular components from capillary-bound Cryptococcus neoformans via endothelial nucleotide signaling
Source: PLoS Biol. 2026 Feb 6;24(2):e3003642. doi: 10.1371/journal.pbio.3003642 (PMC12904584; doi:10.1371/journal.pbio.3003642)
Supplement: S1 Table — (PDF) [file pbio.3003642.s010.pdf]

S1 Table. The information of mutant genes used in the study

Gene symbol[a]	CNAG#	Reference PMID[b]	Gene annotation or function	Results from literature[c]
ada2	CNAG_01626	22174677	transcriptional adapter 2-alpha	required for growth and virulence (i.n.)
ade2	CNAG_02294	8406836	phosphoribosylaminoimidazole carboxylase	avirulent in central nervous system
cap59	CNAG_00721	15075268	alpha-1,3-mannosyltransferase	acapsular and avirulent
cap60	CNAG_00600	9573112	capsule associated gene	acapsular and avirulent
cap64	CNAG_02885	8675296	capsule associated gene	acapsular and avirulent
cfo1	CNAG_06241	19700638	acidic laccase	increased survival (i.n.)
cft1	CNAG_06242	18282105	Cryptococcus ferric transporter 1	attenuated for virulence (i.v.)
cig1	CNAG_01653	23322859	cytokine inducing-glycoprotein	contribute to virulence conditionally
cps1	CNAG_04320	17545316	polysaccharide synthase Cps1p	reduced interaction with endothelial cells
csr2	CNAG_07636	21784998	chitin synthase/regulator	avirulent in mouse (i.n.)
cuf1	CNAG_07724	17290306	metal-binding regulatory protein CUF1	reduced brain dissemination (i.v.),but no change in lung growth (i.n.)
fbp1	CNAG_05280	21478432, 24478071	F-box and leucine-rich repeat protein GRR1	avirulent in mouse (i.n.)
gat201	CNAG_01551	21402362	GATA family transcription factor	attenuated for virulence (i.n.)
gat204	CNAG_06762	21402362	GATA transcription factor, capsule-independent antiphagocytic protein	required for lung colonization (i.n.)
gcs1	CNAG_05583	21283686	ceramide glucosyltransferase	avirulent in mouse (i.n.)
ire1	CNAG_03670	21852949	IRE protein kinase	reduced brain fungal burden (i.n.)
lac1	CNAG_03465	11500433	laccase	attenuated for virulence
man1	CNAG_04312	11359567	mannose-6-phosphate isomerase	reduced brain fungal burden

mpr1	CNAG_04735	24895304	extracellular elastinolytic metalloproteinase	reduced brain CFU (i.v.&i.n.)
pik1	CNAG_07744	22846723, 20696827	1-phosphatidylinositol 4-kinase	reduced brain fungal burden
pka1	CNAG_00396	11287622	AGC/PKA protein kinase	avirulent, failure to produce melanin or capsule
plb1	CNAG_06085	9203663, 36786559	phospholipase B	reduced brain fungal burden (i.v.)
ras1	CNAG_01672	10792722	RAS protein (small guanine nucleotide-binding proteins)	temperature sensitive
rim101	CNAG_05431	23322637, 20174553	pH-response transcription factor pacC/RIM101	altered cell surface, no survival difference
rpb4	CNAG_01444	23659661	DNA-directed RNA polymerase II subunit RPB4	reduced brain fungal burden
ssn8	CNAG_00440	21559476	RNA polymerase II holoenzyme cyclin-like subunit	less virulent in mouse (i.v.)
uge1	CNAG_00697	17462022	UDP-glucose 4-epimerase	unable to colonize the brain
ure1	CNAG_05540	10639402, 20424328	urease	reduced brain CFU (i.v.)
uxs1	CNAG_03322	12139628	UDP-xylose synthase	less virulent, all mice survived >90 days

[a] Strains in bold are constructed in house, all others are from the Fungal Genetics Stock Center (FGSC, https://www.fgsc.net).
[b] Not all literature were included.
[c] Different mouse strains or rabbits were used in different studies.
