# Supplementary material for: Microglia sense fungal infections through capsular components from capillary-bound Cryptococcus neoformans via endothelial nucleotide signaling
Source: PLoS Biol. 2026 Feb 6;24(2):e3003642. doi: 10.1371/journal.pbio.3003642 (PMC12904584; doi:10.1371/journal.pbio.3003642)
Supplement: S2 Table — (PDF) [file pbio.3003642.s011.pdf]

S2 Table. The association of microglia with mutant strains.

Strains	CNAG#	microglia association 24 h[a]	microglia association 48 h
H99	-	+++++	+++++
heat killed H99	-	+	+
ada2Δ	CNAG_01626	+++++	+++++
ade2Δ	CNAG_02294	+++++	+++++
cap59Δ	CNAG_00721	+	+
cap60Δ	CNAG_00600	+	+++
cap64Δ	CNAG_02885	+	+++
cfo1Δ	CNAG_06241	++++	+++++
cft1Δ	CNAG_06242	+++++	+++++
cig1Δ	CNAG_01653	+++++	+++++
cps1Δ	CNAG_04320	+++++	+++++
csr2Δ	CNAG_07636	++	+++
cuf1Δ	CNAG_07724	+++++	+++++
fbp1Δ	CNAG_05280	+++++	+++++
gat201Δ	CNAG_01551	++++	+++++
gat204Δ	CNAG_06762	+++++	+++++
gcs1Δ	CNAG_05583	++++	+++++
ire1Δ	CNAG_03670	+++++	+++++
lac1Δ	CNAG_03465	+++++	+++++
man1Δ	CNAG_04312	++	+++
mpr1Δ	CNAG_04735	++++	+++++
pik1Δ	CNAG_07744	+++++	+++++
pka1Δ	CNAG_00396	+	++
plb1Δ	CNAG_06085	++	+++
ras1Δ	CNAG_01672	+++++	+++++
rim101Δ	CNAG_05431	+++++	+++++
rpb4Δ	CNAG_01444	+++++	+++++
ssn8Δ	CNAG_00440	+++++	+++++
uge1Δ	CNAG_00697	+++++	+++++
ure1Δ	CNAG_05540	++++	+++++
uxs1Δ	CNAG_03322	++++	++++

[a] + <20% association, ++ 20%-40% association, +++ 40%-60% association, ++++ 60%-80% association, +++++ >80% association.
