# Supplementary material for: Microglia sense fungal infections through capsular components from capillary-bound Cryptococcus neoformans via endothelial nucleotide signaling
Source: PLoS Biol. 2026 Feb 6;24(2):e3003642. doi: 10.1371/journal.pbio.3003642 (PMC12904584; doi:10.1371/journal.pbio.3003642)
Supplement: Attachment — Submitted filename: response to reviewers.docx [file pbio.3003642.s025.docx]

IMPORTANT: after discussion with the Academic Editor, we will ask you to address experimentally the requests of Reviewer 1, especially regarding the high dose. Therefore the experiments suggested with lower dose or the survival curves to provide context will be important for publication.

Thank you for offering us the opportunity to revise and improve our manuscript. We have performed the required experiments, and believe that we have addressed the reviewers comments.

Reviewer #1: 

The authors report that brain-resident microglia vigilantly detect Cryptococcus neoformans within capillaries prior to its transmigration across the blood-brain barrier, but are less responsive to non-encapsulated fungi or parenchymal injections of C. neoformans. They further propose that the fungal capsule activates endothelial cells to release nucleotides, which then signal through microglial P2Y12. These are interesting and potentially important findings. However, I have several concerns outlined below.

We thank the reviewer for his/her interest in our story.

Main Concerns
* The figures give the impression that too many microscopic images are crammed together, which sometimes distracts from the main narrative. The manuscript would benefit from streamlining by moving less essential images to supplementary figures and highlighting only the most important data in the main figures.

Thank you for pointing out the critical issue, we agree that the previous figures are crammed with microscopic images. In the revised version, we re-designed all the figures to make them look both comfortable and informative. Less essential images like Figs 1I-1K and Figs 5H and 5I in the previous version were moved to supplementary figures.

* Intravenous infection with C. neoformans H99 at 10^7 CFU is considered an "extremely" high inoculum (as supported by multiple prior publications). Such conditions represent a very acute and severe infection. Data on survival, or at least body weight loss, are needed to contextualize the infection severity used in these experiments. This is a great concern, as the mice may be experiencing sepsis-like systemic inflammation, which could trigger widespread responses throughout the body.

We totally understand the concern. 10^7 CFU was used to allow us better perform statistical analysis, but mice with this high dosage begin to die after 3 days (S1K Fig). In the revised edition, we also used a much-reduced inoculum of 1x10^5^ CFU. Mice with this low infection dose can survive more than one week (S1K Fig). We performed 3D imaging to show similar microglia recruitment using this low dosage at very early stage (S2A and S2B Fig). Thus, the microglia recruitment should not be caused by side effects of the high infection dosage.

* Figures 1 and 2 suggest that microglia translocate toward capillaries within 24 hours of infection, with dendritic morphology changes observed in a similar timeframe. However, Neal et al. (PMID: 29162707) reported that microglia were not activated until day 14, based on CD11c and MHC-II flow cytometry. This discrepancy requires explanation. Is it a consequence of the unusually acute infection model? What about microglia located away from capillaries—do they also become recruited toward C. neoformans? A discussion, accompanied by flow cytometry data analyzing CD11c and MHC-II, would be important to address this.

Actually, we don’t think there is discrepancy, because recruitment does not mean activation, especially when measuring CD11c and MHCii. We also confirmed there is undetectable CD11c and MHCii increase after H99 infection after two days, although recruitment is obvious. It is also important to mention the strains used are different, we used H99, and Neal et al, used 52D. The less virulent 52D strain can be controlled at later times, when T cells are involved, whereas the high virulent strain normally kills the host before T cells activation. We added the discussion and relevant figures in the revised manuscript.

Line 474-477: “Full activation of microglia may require the development of T cells [45], which normally takes at least two weeks [46]. Consistent with these studies, although microglia are rapidly recruited, we did not detect significantly increased microglia activation markers at the early stage. ”

* Related to the above, the very high inoculum likely induces a septic-like state with systemic inflammation. This represents a very specific condition that is not typical of most cryptococcal infections seen in clinics. The key question is whether a similar microglial response can be observed under chronic or lower-inoculum infection conditions. Data from lower-dose infections, where mice survive longer than one week, would be valuable.

We totally agree that high dose i.v. infection is not a clinical model. Thus, we used a much lower infection model and confirmed microglia recruitment is still persevered, with the help of 3D imaging. Mice survive more than one week when infected at 10^5, and data showed low dose infected similarly recruited microglia.

See S1K, S2A and S2B Fig.

* The manuscript should provide a clear diagram indicating precisely which brain regions were evaluated in the imaging analyses.

We provided an illustration showing the regions of brain analyzed as in the new Fig 1E. For quantification of fungal-microglia interaction in the brain, the brains were sectioned into 9-10 coronal slices of 1 mm thickness, and the 3-7th slice from anterior to posterior were analyzed. Notably, we found the response of microglia was rather homogenous throughout the brain.

Line 131-133: “The brains were sliced into 1 mm coronal sections and the 3-7th slice from anterior to posterior were imaged after tissue clearing for quantification (Left).” See updated Fig 1E.

* Line 186 states that "microglia recruitment is strongly associated with vascular leakage." However, lectin staining delineates vascular structures and does not directly measure leakage. This needs clarification.

Yes, we agree lectin staining delineates vascular structures, but since we treated mice with overdosed labeled lectin, there should be abundant unbound free lectin in the circulation which leaked outside the capillaries. We added the clarification in the revised manuscript.

Line 215-216: “Since an overdose of lectin was used, unbound free lectin in the circulation may leak outside the capillaries.”

* The authors show that microglia recognize C. neoformans in capillaries. Do microglia also recognize the pathogen once it has entered the parenchyma, away from vessels? This point remains unclear.

We found microglia is highly responsive to C. neoformans in the capillaries. For those fungi entered the parenchyma, microglia response is much reduced, although some association can occasionally be detected like in S3B Fig. Moreover, if we directly inject C. neoformans into the parenchyma, the microglial response is rather weak (S3B Fig). We added this to the discussion.

Line 549-551: “Supporting this concept, microglial responses are much reduced once *C. neoformans* has entered the parenchyma as shown in S3A and S3B Fig.”

Verbiage Issues
* The term "interaction" between microglia and C. neoformans may be overstated, since the data primarily show proximity rather than direct interaction. A more precise term should be used.

Thanks for pointing out, we agree the term “interaction” may be misleading. We used “recruitment” or “association” instead of “interaction” in the revised manuscript to avoid confusion.

* The repeated use of "parenchyma-residing macrophages" is confusing, since the manuscript focuses on microglia. The terminology should be clarified.

Thanks, we replaced repeated “parenchyma-residing macrophages” to microglia.

* In Figure S3F and related analyses, the evaluation of fungus-host cell "proximity" based on "fungal localization" is unconventional. The authors should explain why this approach was chosen.

In figure S3F, as a supplementary approach to our imaging results, we further used flowcytometry to study the association of fate of Cn (whether they are free or associated with immune cells). We have to gate Tdtomato+ Cn first, because the number of Cn is very low compared to the larger number of immune cells, especially microglia in the brain.

* Figure 2C: The y-axis is labeled "% of all C. neoformans with microglia," and one group is labeled "Within capillary." While the intent is clear (fungi within the capillary), the current wording could be misread as suggesting that microglia are inside capillaries. More precise labeling is needed.

Thanks for pointing out, we changed to “intravascular Cn” and “extracellular Cn” in the revised version.

See updated Fig 2C.

* Figure 4F: The y-axis is also labeled "% of C. neoformans with microglia," but data from many groups presented are from peripheral organs. This labeling is confusing and should be revised.

Sorry for pointing out the mistake, it should be “CFU” of different organs, we corrected in the revised version.

See updated Fig 4F.

 
Minor Points
* For Figure 1J, K, include representative images from each timepoint in the supplementary figures.

We modified as suggested by moving the representative images to supplementary figures.

* Line 135: "Interestingly, although microglia in the cerebellum typically exhibit longer   dendrites than those in the cortex (Fig. S1F), they are equally responsive to fungal invasion." The meaning of this sentence is not clear: Why are they "equally response" when they show longer dendrites?

The longer dendrites are intrinsic features of cerebellum microglia at naïve conditions. These cerebellum microglia are also responsive to *C. neoformans* similar to cortex microglia.

* Figures 5B, I: Capsule thickness was inferred from spacing between stains. Why was capsule staining not performed directly with a GXM-specific antibody? Direct capsule labeling would strengthen the conclusions.

Most of our imaging are performed by 3D imaging after tissue clearing, not traditional immunofluorescence by staining tissue slices. Although the GXM antibody staining on 3D sample is theoretically doable, we did not try because the cost is too high for us.

------------------------------------


Reviewer #2:

This is an interesting manuscript on the role of microglial cells in cryptococcal CNS infection.  This study reveals that brain-resident microglia can detect the fungus Cryptococcus neoformans before the yeast crosses the blood-brain barrier. Using advanced in situ imaging, the authors demonstrate that microglia rapidly migrate to and enwrap capillaries containing viable C. neoformans, an effect absent with nonencapsulated or dead fungi. Rather than killing the fungus, microglia uptake promotes fungal proliferation. The response is triggered not by direct fungal contact but by release of the Cryptococcus capsule polysaccharide glucuronoxylomannan (GXM) (i.e. CrAg), which activates endothelial cells to release nucleotides sensed by microglial P2Y12 receptors. Genetic and pharmacologic blockade of P2Y12 reduced microglial recruitment and fungal growth. These findings define a new paradigm of pathogen sensing by parenchymal macrophages—via endothelial signaling rather than
direct contact.    The authors suggest that modulating microglial activation may represent a therapeutic avenue against Cryptococcus. This last point is fanciful and impractical at present, based on the timing of when actual humans with cryptococcal meningitis present, but the authors are entitled to their opinion. The experiment of nature (i.e. reality), suggests that Th1 CD4 cells are critically important for control of Cryptococcus as absence of CD4s (e.g. HIV) is the global #1 risk factor.

Overall, this is well written, and I have no substantial comments.

Thank you very much for your support!
------------------------------------


Reviewer #3 (Rachael Dangarembizi):

This manuscript investigates the recruitment of microglia to C. neoformans trapped in brain capillaries and examines how microglia interact with the fungus before it crosses the blood-brain barrier. The study demonstrates that microglia detect pathogens indirectly via endothelial cell-derived danger signals triggered by fungal capsular components, particularly GXM. This represents a meaningful addition to our understanding of neuroimmune sensing and inflammatory signaling during cryptococcal infection. The manuscript is well written, and the authors present elegant spatial and temporal imaging data that illuminate a signalling axis in host-pathogen communication that has been largely unresolved in the field.

We are grateful for the supportive words!

My main questions/concerns are:
1. The authors suggest a GXM-related recruitment of microglia which depends on the release of ATP by endothelial cells but the link between these events is not explained (Is ATP being released by damaged endothelial cells? The suggestion seems to be that the endothelial cells are intact. Alternatively, does ATP release by endothelial cells trigger cytokines/chemokines that recruit microglia? Or does ATP itself directly attract microglia? A causal link is necessary to helps readers understand the proposed signaling paradigm.

Thanks for the suggestion! Our in vitro study showed that GXM is sufficient to induce ATP release from endothelial cells, without causing obvious cell damages as indicated by cell viability assay. Thus, ATP release is unlikely from endothelial damage or death.

We showed fungal GXM induces endothelia ATP release and nucleotide receptor P2Y12 is involved in microglia recruitment. However, the exact role of ATP and how it acts on P2Y12 are still elusive. Previous studies have shown that ATP is not a chemoattractant itself, but can promote chemotaxis via autocrine/paracrine fashion (PMID: 22057273, PMID: 20947763). Thus, we suggest that ATP is likely playing an amplification role during microglia recruitment. At this point, the exact chemokines mediating microglia recruitment also need further study. An in vitro study showed fibrinogen and albumin may be the chemokines to microglia (PMID: 25500713), however, since both factors are too essential to knockout, plus their receptors are not yet identified, we were unable to confirm their roles during C. n induced microglia recruitment. In addition, whether ATP release by endothelial cells can trigger cytokines/chemokines that recruit microglia is still interesting to study.

We added these limitations to the discussion. Line 497-508.

2. The emphasis of the decrease in CFUs and microglial association with C. neoformans after depletion should be moderated because a number of things change with the depletion including transmigration of fungi from the periphery (which depends on monocytes), altered physiology, signalling, cross talk in the remaining populations, one could argue that they are technically not in the same state as normal microglia. PLX3397's systemic and cellular effects should be acknowledged.

Thanks for pointing out this issue, we agree that PLX3397 may have profound roles. However, the Cx3CR1cre DTA depletion may further support the conclusion. In fact, our data is also consistent with a recent study showing microglia is not protective during Cn brain infection. We added this point to the revised version to ensure scientific rigor.

Line 288-290: “Notably, PLX3397 may have profound systemic and cellular effects, especially its monocyte inhibitory roles. To independently validate these findings, we also employed a genetic microglia depletion model using CX3CR1CreERiDTR mice.”

3. The claim that "nonencapsulated fungi fail to elicit robust microglial responses" is not fully supported, since the comparison is made across different fungal species with distinct morphologies, PAMP profiles, and pathogenic strategies (S. cerevisiae, C. albicans). A more appropriate comparison would be between encapsulated and nonencapsulated strains of C. neoformans, where the PAMP repertoire is conserved and capsule deficiency is the main variable. Incorporating or highlighting the data from acapsular C. neoformans mutants would strengthen the conclusion and avoid overgeneralisation.

We understand the concern, and now compared the encapsulated and well-known nonencapsulated strains of *C. neoformans* including cap59Δ, cap60Δ and cap64Δ, and showed these strains have dramatically reduced microglia recruitment.

Line 314-319: “Capsule production in C. neoformans is governed by several capsule-associated genes, among which, cap59Δ, cap60Δ and cap64Δ are the first identified. We confirmed the lack of capsule for these mutants in vivo (Fig 5A and 5B). Notably, all three mutants showed impaired microglia association (Fig 5C and 5D), with cap59Δ elicited the weakest response followed by cap60Δ, and cap64Δ showed the strongest response among them (Fig 5D and Movie S6-S8).”
